# Identification and Characterization of a Novel Nontranslated Sequence Variant of the Human Intestinal Di-/Tripeptide Transporter, *hPEPT1*

**DOI:** 10.1155/2012/743472

**Published:** 2012-12-30

**Authors:** Helle Bach Søndergaard, Carsten Uhd Nielsen, Birger Brodin

**Affiliations:** ^1^Department of Pharmaceutics, The Faculty of Medicines and Health, University of Copenhagen, Universitetsparken 2, DK-2100 Copenhagen, Denmark; ^2^Danish Multiple Sclerosis Research Center, University Hospital Rigshospitalet, Blegdamsvej 9, DK-2100 Copenhagen, Denmark

## Abstract

The human H^+^-coupled di-/tripeptide transporter (hPEPT1) mediates intestinal absorption of dietary di- and tripeptides, as well as several peptidomimetic drug compounds. The aim of the present study was to investigate the possible role of the hPEPT1 variant hPEPT1-RF in hPEPT1 regulation. However, the proposed *hPEPT1-RF* mRNA sequence could not be detected in Caco-2 cells or in human intestinal samples. Instead, a new sequence variant, *hPEPT1-RFI*, was found, which is almost identical to the proposed hPEPT1-RF, except for two nucleotide insertions and one deletion that resulted in a changed open reading frame as compared to *hPEPT1-RF*. *In vitro* translation analysis showed that *hPEPT1-RFI* was not translated. In conclusion, the existence of hPEPT1-RF could not be confirmed; furthermore, the identified sequence variant, *hPEPT1-RFI*, does not appear to be translated and is therefore unlikely to have a regulatory effect on hPEPT1 transport activity.

## 1. Introduction

The human *SLC15A1* gene codes for the proton-coupled di-/tripeptide transporter hPEPT1. hPEPT1 mediates small intestinal absorption of dietary di- and tripeptides and a number of peptidomimetic drug compounds (see [1]). A putative splice variant of the *SLC15A1* gene, the hPEPT1-regulatory factor (*hPEPT1-RF*), has been described [2]. *hPEPT1-RF* was found by screening a human duodenum cDNA library with a *hPEPT1* cDNA probe. The cDNA clone was 1724 bp and encoded a putative 208-amino-acid protein. The hPEPT1-RF protein shifted the pH optimum of hPEPT1-mediated peptide uptake slightly towards higher pH values, when hPEPT1 and hPEPT1-RF were coexpressed in Oocytes [2]. Using bioinformatics, the *hPEPT1-RF* sequence was shown to consist of 6 exons, three of which were completely shared with *hPEPT1* mRNA and two partially shared [3]. No studies on the mechanism of the proposed hPEPT1-RF mediated hPEPT1 regulation are available. A few studies have addressed the mRNA expression of *hPEPT1-RF *in Caco-2 cells and human intestinal biopsy samples [4, 5]. In the present study we tried to generate a construct of *hPEPT1-RF* from Caco-2 cells by PCR cloning. However, no *hPEPT1-RF* mRNA was detected. Instead, a novel mRNA sequence variant was identified, which we showed is transcribed in human intestinal tissues and Caco-2 cells, although at low levels. The novel mRNA sequence was highly similar to the putative *hPEPT1-RF* in the initial parts of the sequence but had a new open reading frame (ORF). We named this novel sequence variant human peptide transporter-regulatory factor inactive (hPEPT1-RFI) since western blotting, confocal laser scanning microscopy (CLSM), and *in vitro* translation analysis showed that *hPEPT1-RFI *mRNA was not translated. The hPEPT1-RFI protein is therefore not likely to be a regulator of hPEPT1 transport activity at the protein level.

## 2. Methods

### 2.1. Cell Culture

Human embryonic kidney (HEK 293) and human colon adenocarcinoma (Caco-2) cells were obtained from the American Tissue Culture Collection (ATCC). HEK 293 and Caco-2 cells were grown at 37°C, in an atmosphere of 5% CO_2_ in Modified Eagle Medium (MEM) and Dulbecco's Modified Eagle Medium (DMEM) media, respectively (Gibco, Invitrogen), supplemented with 10% fetal calf serum (FCS), 1% L-glutamine, 1% nonessential amino acids, 100 U/mL penicillin, and 100 *μ*g/mL streptomycin. The HEK 293 MEM media contained 1% sodium pyruvate.

### 2.2. Vector Construction

A PCR segment of 1041 bp containing the *hPEPT1-RFI* sequence was used for vector construction. The segment included the nucleotides ranging from −181 to +847 [gene accession: **AL353574**], an extra 5′-stabilizing G, and recognition sites for the restriction enzymes *Nhe*I and *Xho*I (New England Biolabs). Amplicons were obtained by PCR on cDNA obtained from Caco-2 cells using the primers [5′- G*GCTAGC*AGTTACTGAATCTGCGTTGG] (sense) and [5′-C*CTCGAG*AGTCACAGAAATATCACTGGG] (antisense). The *Nhe*I and *Xho*I digested PCR amplicon containing the coding region without stop codon was cloned into the pcDNA3.1(B) *myc*/His vector (Invitrogen) in reading frame with the *myc*/HIS tag according to manufacturer's instructions. The presence of the *hPEPT1-RFI *transcript was subsequently verified by PCR on DNase treated mRNA isolated from HEK293 cells. Full-length *β*-*ACTIN* was PCR cloned and used for the real-time PCR standard curve. A segment of 1233 bp (−55/+1164) [gene accession: **BC004251.1**] covered the coding sequence. 

PCR reactions were performed in a Thermo cycler (MJ Research, PTC-200, Peltier Thermal Cycler), using a PCR kit from ABgene and the *PfuTurbo* Hotstart proofreading polymerase for amplification (Stratagene). PCR products were separated on 1.0% GTG Seaplaque agarose gels (Cambrex) and analyzed on a Kodak Image Station 1000. Cloning inserts were sequenced on sense and antisense strands (Eurofins MWG operon, DE). 

### 2.3. DNA Transfection

HEK 293 cells were plated two days prior to transfection on 12-well plates (Sigma-Aldrich) coated with 33 *μ*g·mL^−1^ of polylysine, at a density of 7.95 × 10^4^ cells/well. Transfections were performed at 90% confluency, using 4 *μ*L Lipofectamine (Invitrogen) per well, according to the manufacturer's instructions. Transfection media were exchanged with supplemented MEM media four hours after transfection. Cells were lysed 48 hours following transfection and used for western blotting. An estimate of transfection efficiency of HEK 293 cells was done by estimating HEK 293 cells with eGFP-containing plasmids. The eGFP transfection efficiencies were between 40% and 50%. 

### 2.4. Quantitative Real-Time PCR Analysis. 

*PEPT1-RFI* and *PEPT1* mRNA from Caco-2 cells and human cDNA samples from human jejunum (BioChain, AMS Biotechnology) were quantified using real-time PCR analysis. Caco-2 cell RNA was isolated using the RNeasy kit (Qiagen) according to the manufacturer's instructions. Samples were treated twice with RNase free DNase I to remove genomic DNA. Only RNA with an absorbance ratio of 1.8–2.0 as measured by the 260/280 nm ratio was used. Reverse transcription was performed on 0.5 *μ*g RNA with an anchored oligo(dT)primer for first strand cDNA synthesis using the R*everse-i*T kit (ABgene), using *β*-actin as an endogenous control. *β*-actin expression levels in Caco-2 cells were not different between 7 and 24 days in culture (mean *C*_*T*_-values ± SD: 16.14 ± 0.18 and 16.30 ± 0.09, resp.). PCR amplification of *hPEPT1-RFI* was performed by using the primers [5′-TGCTTCCTTCCCTGTGAGTT] (sense) and [5′-AGATGGATGCCCATGCTAAG] (antisense) resulting in an amplicon length of 104 bp. The primers used for *hPEPT1* were [5′-TTGATGTAAACAAACTGACAAGGA] (sense) and [5′-ACTAGAAGCGTGTGGCGTTG] (antisense) resulting in an amplicon length of 103 bp, and the primers for PCR amplification of *β*-actin were [5′-ATTGCCGACAGGATGCAG] (sense) and [5′-CGATCCACACGGAGTACTTG] (antisense) resulting in an amplicon length of 100 bp. All primers used were from DNA technology (Aarhus, DK). Real-time PCR amplifications were performed using an ABI GeneAmp 5700 (Applied biosystems, DK), in a total volume of 50 *μ*L, containing 1 *μ*L cDNA sample, 0.2-0.3 *μ*M of each primer and 1 × SYBR green PCR Master Mix (Applied Biosystems). Reactions were performed in triplicate and nontemplate controls were included. Reaction parameters were 10 min at 95°C, followed by 39 cycles of amplifications (20 s at 95°C and 1 min at 60°C), and ending with a dissociation curve. Plasmid standards were constructed by PCR cloning of the targets, as well as the pcDNA3.1[*hPEPT1*] plasmid (provided by Dr. Wolfgang Sadee, Ohio State University, Columbus OH, USA). Standards were prepared with 10-fold serial dilutions and included in triplicates in each run. Results were normalized to the endogenous control and the fold difference between two targets was obtained by dividing with a calibrator sample. Real-time PCR quantification was performed by using the relative standard curve method, and results are presented as means of the normalized target values relative to the calibrator sample ± standard deviation, as calculated using the relative standard curve method [http://www.appliedbiosystems.com/]. 

### 2.5. Restriction Endonuclease Analysis

To verify that the cDNAs obtained from human were identical to the *hPEPT1-RFI* sequence from Caco-2 cells, we designed a restriction endonuclease assay to distinguish between the presence or absence of a T (848Tdel), where absence of the T indicates that the RNA transcript is the *hPEPT1-RFI* transcript. A PCR amplicon of 104 bp (generated by the same primers as used in the quantitative real-time PCR) was generated from reverse-transcribed DNase I treated RNA from Caco-2 cells grown for 24 days, human jejunum (BioChain, AMS Biotechnology), and ileum and colon. The ileum and colon sample was a gift provided by Dr. Jesper T. Troelsen (Panum Institute, University of Copenhagen, DK). The restriction endonuclease *Mnl*I (NEB) recognizes the target sequence and an internal restriction site, generating two fragments of 34 bp and 70 bp for the *hPEPT1-RFI* amplicon and three fragments of 23 bp, 34 bp, and 47 bp for the *hPEPT1-RF* amplicon. The digested PCR amplicons were run on a 3% MetaPhor agarose gel (Cambrex). 

### 2.6. Confocal Laser Scanning Microscopy Analysis

Cells were fixed and permeabilized as described previously [8]. Cellular expression of *myc*/HIS tagged hPEPT1-RFI was assessed using an anti-*myc*-FITC antibody (1 : 500) (Invitrogen). Expression of the hPEPT1 protein was assessed by the primary anti-hPEPT1 IgG antibody (1 : 1,000), followed by labeling with alexa 488-conjugated goat-anti-rabbit IgG antibody (8 *μ*g/mL). Nontransfected, mock-transfected, and hPEPT1-transfected cells were used as controls. 

### 2.7. Western Blot Analysis

A peptide, identical to the last 13 C-terminal amino acids of the suggested hPEPT1-RF protein sequence, was synthesised (GKRYLKGTSVGLF) (Shafer-N, DK). The epitope is also present in the predicted hPEPT1-RFI amino acid sequence. A polyclonal anti-hPEPT1-RF/RFI antibody was raised in rabbits (Department of Experimental Medicine, University of Copenhagen, DK) and affinity purified. High affinity binding to the epitope was verified by ELISA (data not shown). 

hPEPT1-RFI protein expression in transiently transfected HEK 293 cells was investigated using a mouse anti-HIS antibody. HEK 293 cells or Caco-2 cells were lysed in a buffer containing 2% NP40 buffer, as described previously [8]. The boiled samples, each containing 10–14 *μ*g protein, and 250 ng of a positive control protein of the histidine tag (Positope, Invitrogen), were run on a 4%–20% duramide PAGEr tris-glycine gel (Cambrex), together with the markers MagicMark and SeeBlue (Invitrogen). The gel was transferred to a nitrocellulose membrane (Bio-Rad), blocked in 2% BSA in TBS-T buffer, washed and incubated with a mouse anti-His (C-term) IgG antibody (1 : 5,000) (Invitrogen) or a rabbit anti-hPEPT1 antibody (1 : 1,000) or a rabbit anti-hPEPT1-RF/RFI antibody (1 : 400) diluted in TBS-T with 0.1% BSA. Bound antibody was detected with horseradish peroxidase-conjugated goat-anti-mouse (Medinova) or goat-anti-rabbit (Molecular Probes) IgG antibody and visualized by the ECL Advanced kit (Amersham Biosciences). Chemiluminescence was visualized by a Kodak Image Station 1000. 

### 2.8. * In Vitro* Translation

Translation of the *hPEPT1-RFI* transcript was investigated by coupled *in vitro* transcription and translation using the PROTEINscriptII kit (Ambion, DK), according to the manufacturer's instructions. The transcription reaction was performed with the T7 RNA polymerase on 0.4-0.5 *μ*g of the plasmids; pcDNA3.1[*hPEPT1-RFI*]*myc*/HIS, pcDNA3.1[hPEPT1], pcDNA3.1[mock](negative control), and pTRI-Xef (positive control). A sample without DNA was included. Five micro liters of each translation mix were transferred to reducing sample buffer and boiled for 3 min and run on a 4%–20% duramide PAGEr tris-glycine gel (Cambrex), followed by visualization on a phosphor imager screen (Storm 840, Molecular Dynamics). 

## 3. Results and Discussion

### 3.1. * hPEPT1-RFI * Is a Novel Variant of hPEPT1

**Figure 1:**
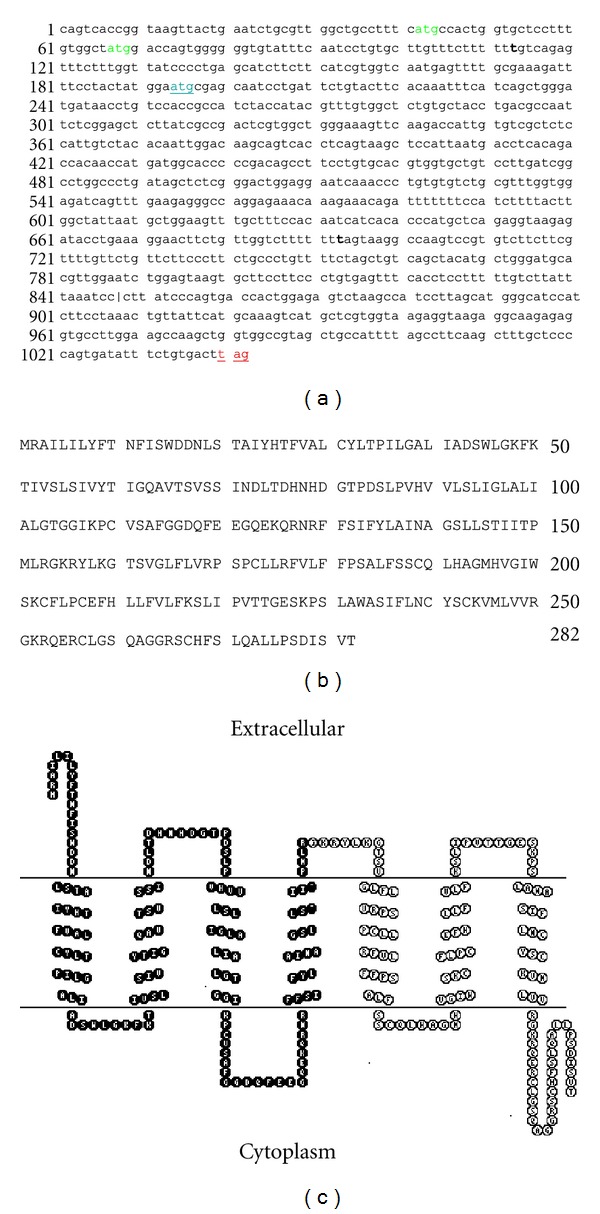
Molecular characterization of human *PEPT1-RFI*; a novel sequence variant. (a) Nucleotide sequence of human *PEPT1-RFI *cDNA from the 5′untranslated region to the deduced *hPEPT1-RFI* stop codon. The insertions 113Tins and 693Tins (marked in bold) and one deletion (848Tdel) (marked by a line) separate this sequence from the *hPEPT1-RF* sequence. Deduced start and stop codons are underlined. (b) Putative amino acid composition of hPEPT1-RFI [accession number **AL353574.8**]. (c) Topographic orientation of the hPEPT1-RFI protein as predicted by the HMMTOP transmembrane topology prediction server [6, 7]. The hPEPT1-RFI corresponds to hPEPT1 in the sequence shown in black circles. The image was generated using TOPO2, transmembrane protein display software (Johns S. J., http://www.sacs.ucsf.edu/TOPO2/).

Initially we attempted to isolate mRNA for the proposed regulatory factor of hPEPT1, *hPEPT1-RF*, from Caco-2 cells by PCR cloning. However, it was not possible to identify a sequence corresponding to the proposed *hPEPT1-RF* sequence. We discovered, however, a novel sequence with great similarity to the published *hPEPT1-RF* sequence, but with three nucleotide changes. This novel sequence variant of *hPEPT1* was named *hPEPT1-RFI*. The sequence carried two insertions (113 Tins and 693 Tins) and one deletion (848Tdel) as compared to *hPEPT1-RF* (accession number [Genbank: **AB001328.1**]) (Figure 1(a)). The identity of this sequence variation was confirmed by sequencing, in both directions, of two independently generated *hPEPT1-RFI* inserts cloned from Caco-2 cells. The *hPEPT1-RFI* sequence had a different open reading frame (ORF) as compared to *hPEPT1-RF*. The sequence yielded a predicted protein of 282 amino acids with a calculated molecular weight of ~31 kDa (30,909 Da). The first 153 amino acids (Figure 1(b)) are identical with hPEPT1, while the last 129 amino acids are unique. Topology analysis by the HMMTOP transmembrane topology prediction server [6, 7] suggested a protein with 7 transmembrane segments (TMS) of which the first four TMS are identical with TMS 2–5 of hPEPT1 (Figure 1(c)). ORF finder searches on the genomic clone that contains the *SLC15A1* sequence revealed a 282-amino-acid ORF corresponding to *hPEPT1-RFI* [GenBank: **AL353574.8**], supporting the existence of the *hPEPT1-RFI* sequence. The presence of an ORF encoding *hPEPT1-RFI* in the *SLC15A1* sequence thus indicates that *hPEPT1-RFI* can be generated by the existing sequence, but we cannot at present explain the lack of expression of *hPEPT1-RF*. 

### 3.2. * hPEPT1-RFI * mRNA Is Found in Both Caco-2 Cells and Tissues from Healthy Humans

**Figure 2:**
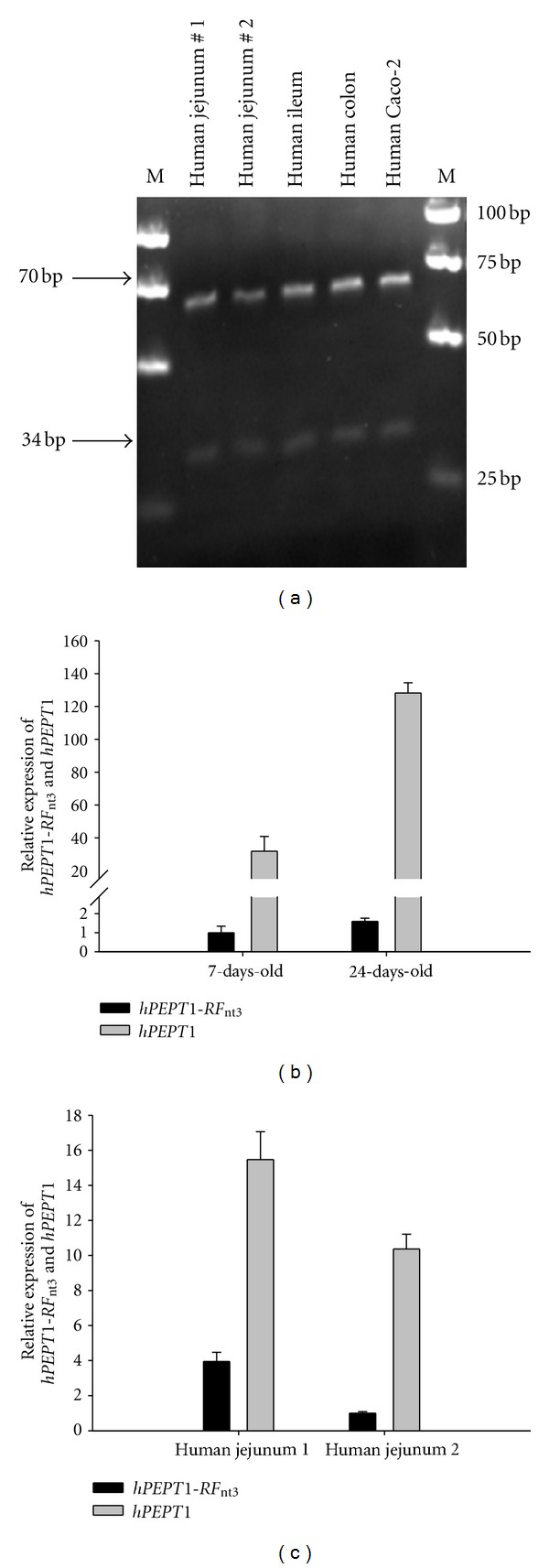
*hPEPT1-RFI* mRNA is expressed in Caco-2 cells and human tissues. (a) The deletion 848delT in the *hPEPT1-RFI* sequence was used to differentiate between the *hPEPT1-RFI* and the *hPEPT1-RF* transcripts. Absence of the T indicates that the RNA transcript is the *hPEPT1-RFI* version. RT-PCR was performed on human jejunum, ileum, colon, and Caco-2 mRNA (Lane 1–5), and amplicons were digested by the restriction endonuclease *MnlI* and analysed by agarose gel electrophoresis. The arrows indicate a 34 bp and a 70 bp segment in concordance with the *hPEPT1-RFI* sequence. M denotes the molecular weight DNA ladder. (b) Real-time PCR was performed with primers covering parts of *hPEPT1-RFI*, *hPEPT1*, and *β*-*ACTIN* as described in methods. RNA was sampled from Caco-2 cells grown for 7 and 24 days. Reverse transcription was performed on 0.5 *μ*g RNA, and results are presented as means of normalized target values relative to a calibrator sample ± SD of three individual experiments, each performed in triplicates. (c) Real-time PCR was performed on human intestinal cDNA from two healthy individuals. The sample RNA was taken from the jejunal part of the intestine. Results are presented as means of normalized target values relative to the calibrator sample ± SD of each determination in triplicate.

To investigate if the *hPEPT1-RFI* and/or the *hPEPT1-RF* sequences are transcribed in human tissues, we constructed a restriction endonuclease assay. This assay cuts the PCR amplicon sequence at a control site and at a second site that is only present in the *hPEPT1-RF* sequence (848T). Thus, appearance of two bands in the gel identifies the *hPEPT1-RFI* sequence. PCR amplicons were generated from cDNA samples obtained from human jejunum, ileum and colon, and from Caco-2 cDNA, as a control, and digested by the restriction endonuclease *Mnl*I. The result of the restriction enzyme analysis showed that all of the investigated amplicons had the 848Tdel mutation, which is present in the *hPEPT1-RFI* sequence (Figure 2(a)). Furthermore, the human adenocarcinoma cell lines OVCAR-5 and SK-OV-3 contained the 848Tdel mutation, the human glioblastoma cell line, SNB-19, and the colon carcinoma cell line HCT-116 (results not shown). These results indicate that the *hPEPT1-RFI* sequence and not *hPEPT1-RF* seems to be present in all tissues tested. 

### 3.3. *hPEPT1-RFI* mRNA Is Expressed at Low Levels in the Caco-2 Cell Line and in Human Intestinal Tissues

Real-time PCR was performed on reverse transcribed mRNA from Caco-2 cells at passage 23 sampled on days 7 and 24 of growth (Figure 2(b)). The relative expressions of *hPEPT1-RFI* and *hPEPT1* mRNA in Caco-2 cells were 1.0 ± 0.3 and 31.9 ± 9.0 on day 7 and 1.6 ± 0.2 and 128.2 ± 6.3 on day 24, respectively (*n* = 3). Thus, *hPEPT1-RFI* showed a low mRNA expression profile in Caco-2 cells relative to *hPEPT1*. Furthermore, only a minor increase in *hPEPT1-RFI* expression during 24 days of cell growth was seen compared to the *hPEPT1* expression pattern for the same time period. In human tissues, the *hPEPT1-RFI* and *hPEPT1* expressions were investigated on cDNA from the jejunal part of the small intestine, from two healthy individuals (Figure 2(c)). The relative expressions of *hPEPT1-RFI* and *hPEPT1* mRNA in the first individual were 3.95 ± 0.5 versus 15.5 ± 1.6 and in the second individual 1.00 ± 0.09 versus 10.4 ± 0.8, respectively. From the total *hPEPT1*-related transcripts 20% of these were of *hPEPT1-RFI* origin in the first individual and 9% were of *hPEPT1-RFI* in the second individual, indicating that the *hPEPT1-RFI* transcript is expressed at low levels compared to *hPEPT1* and that the ratio between *hPEPT1* and *hPEPT1-RFI* of the total *hPEPT1*-related transcripts shows high variation in the examined human tissues. Anderle et al. (2006) showed that *hPEPT1-RF* mRNA from human intestinal biopsies contributed with 2%–44% of the total *hPEPT1*-related transcripts [5]. In this study PCR was performed on *hPEPT1*-related transcripts, and the three nucleotide changes between *hPEPT1-RF* and *hPEPT1-RFI* may not have been noticed, or in other words, it seems possible that the detected mRNA was *hPEPT1-RFI* rather than *hPEPT1-RF*. 

### 3.4. The hPEPT1 Sequence Variant * hPEPT1-RFI * Is Transcribed but Not Translated

**Figure 3:**
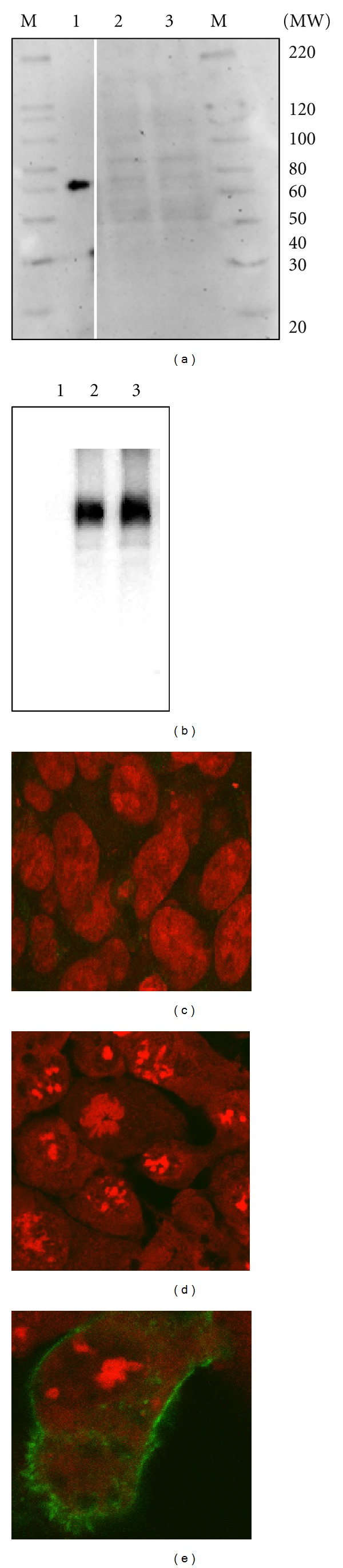
Western blot and immunolocalization analysis of hPEPT1-RFI, hPEPT1, or mock transfected HEK 293 cells. (a) The anti-HIS antibody did not detect any difference between cell lysates from hPEPT1-RFI *myc*/HIS expressing and mock cells (10 and 11 *μ*g) (lane 2 and 3). The positive control Positope protein, containing the HIS epitope, was detected (53 kDa) with the anti-HIS antibody (lane 1). M denotes the molecular weight marker. (b) Western blot on cell lysates from hPEPT1 expressing HEK 293 cells. The anti-hPEPT1 antibody detected the hPEPT1 protein (~80 kDa) (lane 2 and 3) (3 and 10 *μ*g), but as expected not in the mock transfected cells (lane 1) (10 *μ*g). Cells transfections were performed in parallel for the *hPEPT1-RFI*, *hPEPT1*, and mock-containing vectors. (c) Immunostaining of cells transfected with the *myc*/HIS tagged *hPEPT1-RFI*, or mock plasmid (d) using the monoclonal antibody anti-*myc*-FITC antibody (green). Cells were counterstained with propidium iodide to visualize cell nuclei (red). (e) Immunostaining of cells expressing hPEPT1 with an anti-hPEPT1 antibody followed by a secondary alexa 488-conjugated antibody. Immunostaining was completed in two independent cell passages. The image is representative of three individual preparations. Immunostaining against the HIS epitope confirmed these findings in two independent cell passages.

**Figure 4:**
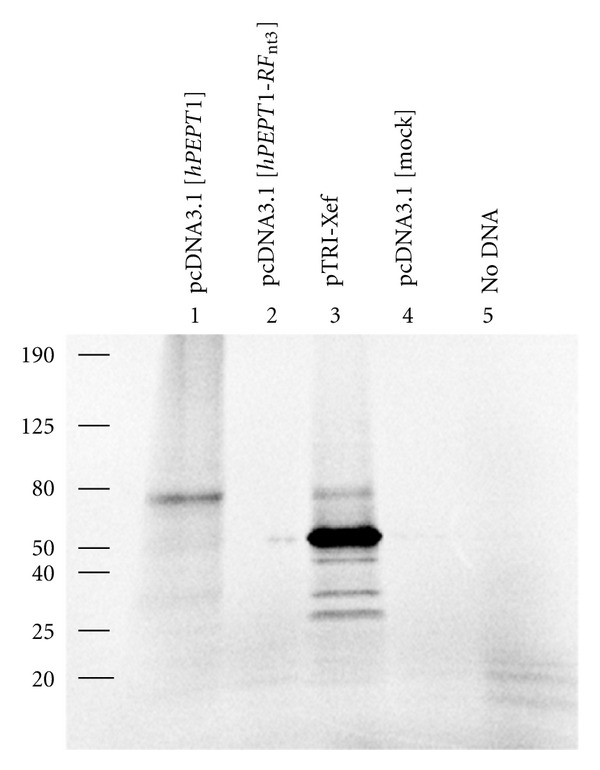
*In vitro* translation of hPEPT1 and TRI-Xef, but absence of hPEPT1-RFI protein. Equal amounts of plasmids with inserts of *hPEPT1-RFI*, *hPEPT1*, TRI-Xef. and empty plasmid were transcribed *in vitro*. Of each transcription mix, 2 *μ*l were used for the *in vitro* translation mixed with reticulocyte lysate. PAGEr Tris glycine gels were exposed to a phosphor imager screen overnight for visualization of the protein-incorporated [^35^S]-Methionine. No protein band was identified in the gel after *in vitro* translation of the *hPEPT1-RFI* containing plasmid (lane 2). Protein bands are shown in the positive controls (lanes 1 and 3) at the expected molecular weights. As expected, negative controls showed no protein bands (lanes 4 and 5).

For investigating if the hPEPT1-RFI protein was present in Caco-2 cells, we used an antibody that was generated against an epitope present in the deduced 282 amino acids of the major ORF. However, this antibody did not show specific epitope staining of any protein band from Caco-2 cells (results not shown). For further investigations, we tagged the *hPEPT1-RFI* with the *myc*/HIS epitope and placed it in front of a strong promoter to be expressed in HEK 293 cells. The protein expression level of hPEPT1-RFI in HEK 293 cells expressing a *myc*/HIS tagged hPEPT1-RFI was investigated using Western blotting. We were not able to detect hPEPT1-RFI protein in any of the tested cell lysates. In protein lysates from HEK 293 cells, no difference could be observed between lanes with *hPEPT1-RFI* and empty (mock) plasmid using the anti-HIS antibody (Figure 3(a)). As controls proteins containing the *myc*/HIS-tag and HEK 293 cells transiently transfected with *hPEPT1* were used (Figures 3(a) and 3(b)). Confocal laser scanning microscopy studies on *hPEPT1-RFI* transfected, and mock transfected HEK 293 cells could confirm the results from the Western blot experiments (Figures 3(c) and 3(d)). hPEPT1 expression in HEK 293 cells was used as a positive control for the transfection procedure (Figure 3(e)). We investigated whether the pcDNA3.1[*hPEPT1-RFI*]*myc*/HIS plasmid could be translated to protein *in vitro*. The positive controls, the pcDNA3.1[hPEPT1] plasmid and the pTRI-Xef plasmid supplied with the kit, gave protein bands at the expected sizes (Lanes 1 and 3, Figure 4) as measured by the incorporation of radioactive labeled methionine. The two negative controls, pcDNA3.1[mock] and-DNA, showed no sign of protein bands. Also, the pcDNA3.1[*hPEPT1-RFI*]*myc*/HIS plasmid showed no protein band (Lane 2, Figure 4). These results, in support of the previous results, showed that no detectable protein is translated from the *hPEPT1-RFI* transcript. Interestingly, Saito and coworkers observed a band of low molecular weight (~23 kDa) after *in vitro* translation of the putative *hPEPT1-RF cRNA* sequence. Possible this band could be an artifact of either radioactive labeled tRNAs, or small polypeptides from the translation of fragments remaining in the reticulocyte lysate. 

In conclusion, we have revealed a novel variant of the *SLC15A1* gene, named *hPEPT1-RFI*. Characterization of this novel sequence showed that it was present in several cell lines of human origin, including Caco-2 cells, and in human intestinal samples. We did not find evidence for the existence of the putative *hPEPT1-RF* sequence, questioning the proposed role of the hPEPT1-RF protein as a regulator of hPEPT1 pH sensitivity. Only a change of three nucleotides separates the *hPEPT1-RF* and *hPEPT1-RFI* sequence, but the impact of two insertions and one deletion was significant, since it changed the reading frame of the *hPEPT1-RF* sequence. Investigations of the putative protein of the *hPEPT1-RFI* sequence by Western blotting, CLSM, and *in vitro* translation gave no detectable results. As a consequence, no protein-protein interaction between hPEPT1-RFI and hPEPT1 can be anticipated.
